# Non-O157 Shiga Toxin–producing *Escherichia coli* Associated with Venison

**DOI:** 10.3201/eid1802.110855

**Published:** 2012-02

**Authors:** Joshua M. Rounds, Carrie E. Rigdon, Levi J. Muhl, Matthew Forstner, Gregory T. Danzeisen, Bonnie S. Koziol, Charlott Taylor, Bryanne T. Shaw, Ginette L. Short, Kirk E. Smith

**Affiliations:** Minnesota Department of Health, St. Paul, Minnesota, USA (J.M. Rounds, B.S. Koziol, C. Taylor, G.L. Short, K.E. Smith);; Minnesota Department of Agriculture, St. Paul USA (C.E. Rigdon, L.J. Muhl, M. Forstner, G.T. Danzeisen, B.T. Shaw)

**Keywords:** Non-O157 Shiga toxin–producing Escherichia coli, Escherichia coli, STEC, bacteria, surveillance, outbreak, foodborne diseases, white-tailed deer, venison, pulsed-field gel electrophoresis, Minnesota, United States

## Abstract

We investigated an outbreak of non-O157 Shiga toxin–producing *Escherichia coli* at a high school in Minnesota, USA, in November 2010. Consuming undercooked venison and not washing hands after handling raw venison were associated with illness. *E. coli* O103:H2 and non-Shiga toxin–producing *E. coli* O145:NM were isolated from ill students and venison.

## Abstract 2

News reports of “E. coli outbreaks” usually refer to Shiga toxin–producing E. coli O157. But there are other types of Shiga toxin–producing *E. coli, *often called STEC, about which less is known. For these other types of STEC, what is the source? What are the risk factors? An outbreak among 29 high school students in Minnesota provided some answers. The source of this outbreak was a white-tailed deer that had been butchered and eaten at the school. The risk factors for infection were handling raw or eating undercooked venison. To prevent this type of STEC infection, people should handle and cook venison with the same caution recommended for other meats.

Non-O157 Shiga toxin–producing *Escherichia coli* (STEC) are emerging pathogens (*1**,**2*) but are underrecognized because relatively few clinical laboratories routinely use culture-independent testing methods necessary for their identification (*3**,**4*). Ruminants (e.g., cattle, goats) can be colonized by non-O157 STEC and are reservoirs of these organisms. Non-O157 STEC outbreaks have been associated with contaminated food and recreational water and with direct contact with infected animals or humans (*2**,**4**,**5*). However, much is still unknown about sources and risk factors for non-O157 STEC infection.

## The Study

On December 1, 2010, the Minnesota Department of Health (MDH) was notified that 2 students from the same high school were hospitalized with bloody diarrhea. As part of a physical education/environmental science class, 7 white-tailed deer (*Odocoileus virginianus*) had been processed on school grounds on November 16, and venison kabobs were grilled and consumed in class on November 23.

MDH and Minnesota Department of Agriculture (MDA) staff interviewed the course instructor and the butcher who processed the deer. The school provided names and contact information for students enrolled in the class. A case–control study was conducted; students were interviewed about illness, food consumption, and venison handling in class. A case-patient was defined as a class enrollee in whom diarrhea (>3 loose stools in 24 hours) developed after November 16 and lasted >3 days. Diarrhea duration was included in the case definition to exclude possible background norovirus infections.

Stool specimens from 6 students were submitted to MDH. Specimens were tested for *E*. *coli* O157 and *Salmonella*, *Shigella*, *Yersinia*, and *Campylobacter* spp. by culture and for norovirus genogroups I and II by PCR. Non-O157 STEC testing was conducted by using sweep PCR for Shiga toxin genes (*stx1* and *stx2*), *hlyA*, and *eaeA* (*6*) and by culture using immunomagnetic separation.

If Shiga toxin genes were detected by sweep PCR but not in isolated colonies, *hlyA*- and *eaeA*-positive colonies were serotyped. Leftover raw venison was tested at MDA for STEC by PCR for *stx1*, *stx2*, and *uidA*; by immunomagnetic separation for STEC O103; and by O145 isolation (*7*). SAS software version 9.2 (SAS Institute, Cary, NC, USA) was used for analyses. p <0.05 was considered significant.

**Figure 1:**
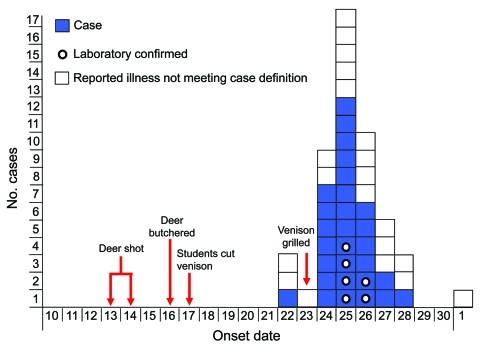
Non-O157 Shiga toxin–producing *Escherichia coli* infections associated with venison among students in a high school class, by illness onset date, November 2010, Minnesota, USA. The case-patient with illness onset on November 22 reported 1 instance of vomiting on that date, followed by a distinct onset of diarrhea on November 24, which suggests that the case-patient may have been co-infected with norovirus and non-O157 Shiga toxin–producing *E*. *coli*.

Of 225 students from 5 class periods, 117 (52%) were interviewed. Twenty-nine case-patients (25%) were identified. Twenty additional students reported gastrointestinal symptoms that did not meet the case definition and were excluded from analysis. Twenty (69%) case-patients were male. Median incubation from the November 23 class date for 28 case-patients with illness onset after that class was 53.5 hours (range 22–121 hours) (Figure 1). All 29 case-patients reported diarrhea, 21 (72%) reported cramps, 5 (17%) vomiting, 5 (17%) bloody stools, and 2 (7%) fever. Median duration of illness was 5 days (range 4–12 days). Two case-patients were hospitalized for 2 and 3 days, respectively. No case-patients showed development of hemolytic uremic syndrome and none died.

**Figure 2:**
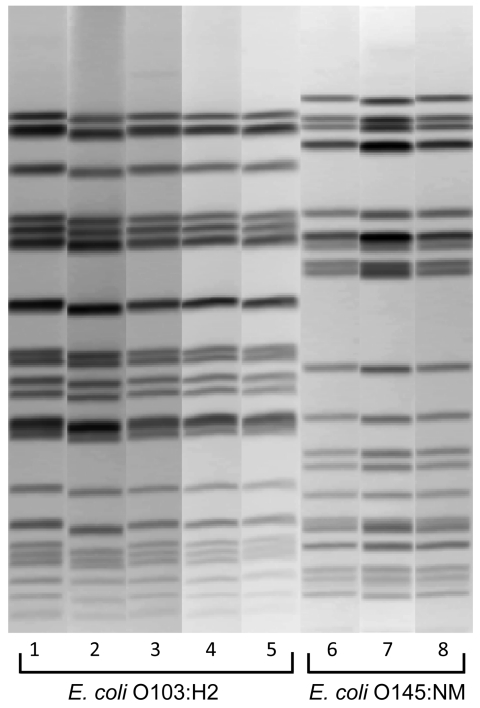
*Xba*I pulsed-field gel electrophoresis patterns of pathogenic *Escherichia coli* from humans and venison, Minnesota, USA, November 2010. Lanes 2, 4, 6, and 8, isolates from venison. Lanes 1, 3, 5, and 7, isolates from human case-patients.

All 6 stool samples were negative for *stx2*, *E. coli* O157, *Salmonella*, *Shigella*, *Yersinia*, and *Campylobacter* spp. Five samples were positive for *stx1*, 5 for *hlyA*, and 4 for *eaeA* by sweep PCR (Table 1). Two of these samples did not yield additional findings. An *stx1*-positive *E. coli* O103:H2 was isolated from 2 samples (from the 2 hospitalized students). Both *E. coli* O103:H2 isolates were indistinguishable by pulsed-field gel electrophoresis (PFGE) (Figure 2). In another sample that was *stx1* positive by sweep PCR, *stx1* was not identified in isolated colonies, but serotyping of *hlyA*- and *eaeA*-positive colonies identified *E. coli* O145:NM (Table 1). A sixth sample was negative for *stx1* and *stx2* by sweep PCR but positive for *hlyA* and *eaeA*; serotyping of *hlyA*- and *eaeA*-positive colonies identified *E. coli* O145:NM (Table 1). Both *E. coli* O145:NM isolates were indistinguishable by PFGE (Figure 2). One of the samples that yielded *E. coli* O145:NM was also positive for norovirus genogroup II.

**Table 1:** Analysis of non-O157 pathogenic *Escherichia coli* from 6 high school students, Minnesota, USA, November 2010*

Case-patient no.	*stx1* sweep PCR	*stx1* isolated colony	*hlyA*	*eaeA*	*E. coli* O103:H2	*E. coli* O145:NM	Norovirus genogroup II
1	+	+	+	+	+	–	–
2	+	+	+	+	+	–	–
3	–	–	+	+	–	+	–
4†	+	–	+	+	–	+	+
5‡	+	–	+	–	–	–	–
6§	+	–	–	–	–	–	–

*+, positive; –, negative. †100 colonies tested; none were *stx1* positive, and 5 were *hlyA* and *eaeA* positive. ‡122 colonies tested; none were *stx1* positive. §Positive enzyme immunoassay results for Shiga toxin at clinical laboratory; *stx*1 positive by PCR of submitted broth. Submitted broth failed to grow *E. coli* colonies.

Six deer were shot and field dressed during November 12–14. A seventh deer was obtained after being hit by a vehicle. Students brought the deer to the school where they were stored in a shed packed in ice. On November 16, a butcher processed each deer by using tools that had never been used to butcher domestic ruminants. Tables, cutting boards, and tools were reportedly cleaned with a 10% bleach solution. Venison was wrapped in plastic, covered in ice, and stored overnight in the shed.

On November 17, students cut selected pieces of meat into cubes, which was wrapped in butcher paper and frozen. Remaining large cuts were returned to students who had provided deer. Students could have received venison from any of the 7 deer. On November 22, the venison was thawed and marinated in 5-gallon buckets. On November 23, several students used wooden bamboo skewers to assemble venison kabobs. The kabobs were grilled by several students on a gas grill for consumption during each class period. Students were instructed to wear gloves and wash their hands after handling raw venison on November 17, 22, and 23.

In the case–control study, consuming undercooked or pink venison was associated with illness (Table 2). Among students who handled raw venison or helped clean up on November 23, students who reported handwashing afterwards were less likely to become ill. Numerous students reported instances of potential cross-contamination or other food handling errors, including using the same plate for raw and cooked venison, using the same tongs to handle raw and cooked venison, and not washing hands after bare-hand contact with raw venison.

**Table 2:** Risk factors for infection with non-O157 Shiga toxin–producing *Escherichia coli* for high school students, Minnesota, USA, November, 2010

Risk factor	No. positive patients/total (%)	No. positive patients/total (%)	Odds ratio (95% CI)
Consumption of undercooked venison	11/26 (42)	11/60 (18)	3.27 (1.18–9.03)
Handwashing*	5/9 (56)	15/16 (94)	0.08 (0.01–0.93)
Wearing gloves†	18/22 (81)	54/56 (96)	0.15 (0.03–0.94)

*Among 25 students who had contact with raw venison or helped clean up on November 23. †Among 78 students who had contact with raw venison during the class on November 17.

Venison butchered at the school and collected from 2 households was positive for *E. coli* O103:H2, which was indistinguishable from the isolates from the 2 case-patients by PFGE. One sample of venison butchered at the school was positive for *E. coli* O145:NM and was indistinguishable from the isolates from the 2 case-patients by PFGE (Figure 2).

## Conclusions

This outbreak of non-O157 STEC was associated with handling and consumption of venison from wild white-tailed deer in a high school class. Venison butchered at the school was positive for the outbreak PFGE subtype of STEC O103:H2 and non-Shiga toxin–producing (stx–) *E. coli* O145:NM.

The role of stx– *E. coli* O145:NM is unknown. Although *E. coli* O145:NM strains isolated from patients 3 and 4 and venison were stx–, other virulence factors, clinical illness, and an enterohemorrhagic *E. coli* serotype suggest a potentially pathogenic strain. Human infections with stx– *E. coli* serotypes may cause bloody diarrhea and hemolytic uremic syndrome (*8*). Further characterization of virulence determinants and phylogeny of these strains may shed light on their pathogenicity.

Multiple potential routes of transmission from venison to case-patients were identified, included consumption of venison and cross-contamination from raw to cooked venison. Handwashing after touching raw venison or contaminated surfaces was protective. Interviews with the butcher ruled out cross-contamination from domestic ruminants to venison during butchering. Therefore, we conclude that >1 deer were colonized with non-O157 STEC.

A study of white-tailed deer feces in Minnesota and Wisconsin found non-O157 STEC in 5% of samples (*9*). Studies have found non-O157 STEC contamination of deer meat ranging from 7.5% of roe deer meat in Germany to 22% of fallow deer meat in Belgium (*5*). Prevalence rates of *E. coli* O157 in deer have ranged from 0.25% to 2.4% (*10**–**12*). Previous outbreak investigations and case reports have linked *E. coli* O157 infections to deer (*13**–**15*). This outbreak indicates that white-tailed deer are a source of human non-O157 STEC infections. Venison should be handled and cooked with the same caution recommended for other meats.
